# Collaborative Task Offloading and Service Caching Strategy for Mobile Edge Computing

**DOI:** 10.3390/s22186760

**Published:** 2022-09-07

**Authors:** Xiang Liu, Xu Zhao, Guojin Liu, Fei Huang, Tiancong Huang, Yucheng Wu

**Affiliations:** 1 School of Microelectronics and Communication Engineering, Chongqing University, Chongqing 400044, China; 2 Beijing Smart-Chip Microelectronics Technology Co., Ltd., Beijing 100005, China; 3 State Grid Chongqing Electric Power Company Electric Power Research Institute, Chongqing 401123, China

**Keywords:** mobile edge computing, collaboration, task offloading, service caching, resource allocation, fairness, load balance

## Abstract

Mobile edge computing (MEC), which sinks the functions of cloud servers, has become an emerging paradigm to solve the contradiction between delay-sensitive tasks and resource-constrained terminals. Task offloading assisted by service caching in a collaborative manner can reduce delay and balance the edge load in MEC. Due to the limited storage resources of edge servers, it is a significant issue to develop a dynamical service caching strategy according to the actual variable user demands in task offloading. Therefore, this paper investigates the collaborative task offloading problem assisted by a dynamical caching strategy in MEC. Furthermore, a two-level computing strategy called joint task offloading and service caching (JTOSC) is proposed to solve the optimized problem. The outer layer in JTOSC iteratively updates the service caching decisions based on the Gibbs sampling. The inner layer in JTOSC adopts the fairness-aware allocation algorithm and the offloading revenue preference-based bilateral matching algorithm to get a great computing resource allocation and task offloading scheme. The simulation results indicate that the proposed strategy outperforms the other four comparison strategies in terms of maximum offloading delay, service cache hit rate, and edge load balance.

## 1. Introduction

With the rapid development of wireless network technology, a large number of computing-intensive and delay-sensitive applications emerge, such as autonomous driving, face recognition, and virtual/augmented reality (VR/AR) [1,2]. The restricted computing performance and storage resources of mobile terminals limit the further development of emerging applications [3,4]. The traditional solution is to offload these application tasks to a cloud server for centralized processing, leading to long transmission time because of its far location [5]. Mobile edge computing (MEC) is an emerging paradigm, which sinks the functions of cloud servers and provides users with required services and computing demands at the edge of network. As an important technology in mobile edge computing, task offloading solves the limitation caused by the insufficient capability of the terminal and relieves core network pressure [6].

As the infrastructure for the extension of cloud services to the edge side, edge servers are required to be modular and miniaturized. To meet the needs of different application scenarios, edge servers should be able to be fully decoupled into computation, storage, communication, management, and other components. Besides, edge servers are designed to be more compact in size. These all limit the resource of edge servers. Compared with powerful cloud servers, the capability gap between them can reach several orders of magnitude [7,8]. When the number of users increases, on the one hand, a single server is not able to support all user tasks, resulting in poor user experience. On the other hand, there is uneven load distribution among multiple edge servers, which causes some edge servers to overload while some to idle. Therefore, it has become a trend for multiple edge servers to perform task offloading collaboratively while considering the computation load balance among edge servers [9,10]. However, these reported works do not consider the limitation of the service caching on task offloading, which will cause the failure of task execution in practical scenarios.

Service caching refers to the cache of program databases and libraries required. Only edge servers with relevant services can execute corresponding user tasks [11]. These services can be downloaded from the remote cloud when user tasks arrive, or they can be cached in MEC beforehand. It will spend tens of seconds temporarily downloading from the cloud server [12]. Therefore, it can effectively reduce the initial delay if various services are cached in advance. Most reported task offloading works in MEC ideally assume that edge servers cache all required services, but the actual edge servers have constrained storage resources and the type of caching services must be chosen wisely [13,14]. Furthermore, the fixed-type service caches are also not suitable for the user with dynamical requirements. Thus, it is necessary to make an efficient and dynamical caching strategy according to the actual task requirements.

In addition, many current works focus on better overall benefits, such as less total delay [15], smaller energy consumption [16], or lower system cost [17]. A solution that only guarantees the overall system benefits may result in unfair treatment of the individual users, which will lead to poor user experience. Hence, fairness among users is also an important issue in MEC [18,19,20].

To solve the problems mentioned above, this paper investigates collaborative task offloading assisted by a dynamical caching strategy, considering user fairness and edge load balance in MEC.

The main contributions of this paper are summarized as follows.

1. We constructed a two-layer collaborative MEC system model. To meet the feasibility constraints of task execution, the services of various emerging applications are dynamically cached in advance at edge servers;
2. To ensure fairness among users to a certain extent, the optimization goal is to effectively reduce the maximum delay of all users. A JTOSC algorithm that comprehensively considers adaptive dynamic service caching, efficient collaborative task offloading, and fair computation resource allocation is proposed;
3. To simplify the solution of the proposed algorithm, JTOSC is decoupled into outer and inner subproblems. The outer layer in JTOSC iteratively updates the service caching decisions based on Gibbs sampling. The inner layer in JTOSC is based on the fairness perception and the offloading revenue preference to get a sensible computing resource allocation and task offloading scheme, respectively. Simulation results have verified the effectiveness of the proposed strategy.

The remainder of this paper is organized as follows. In Section 2, we review the related works. In Section 3, we describe the system model, and the optimizing problem is formulated. In Section 4, we detail the scheme design of joint task offloading and service caching based on edge collaboration. Section 5 evaluates and analyzes the performance of the proposed strategy. Finally, some conclusions for the work are drawn in Section 6.

## 2. Related Works

Currently, task offloading has become a critical issue in mobile edge computing. In [21], an efficient task offloading management scheme in a densely deployed small cell network was studied, using a genetic algorithm and particle swarm algorithm to jointly optimize offloading decision, spectrum resource, transmit power, and computing resource allocation to minimize the energy consumption of users. With the same optimization objective described in [21], multi-users partial computation offloading based on Lyapunov with integrating energy harvesting (EH) technology was presented to achieve long-term operation of the terminal in [22]. The task dependency model for multiple users was considered in [23], which focused on addressing the combination of offloading decisions among tasks and the strong coupling with resource allocation to minimize the weighted sum of energy consumption and delay for users. It was pointed out in [24] that cooperation among MECs could yield huge performance gains while balancing the computational load. From the perspective of game theory, efficient vehicle task offloading was achieved through thermal-aware MEC collaboration based on the analysis of vehicle users running trajectories to reduce the task completion delay significantly in [25]. The horizontal cooperation of multiple MEC-BSs was proposed to further offload additional tasks to the remaining MEC-BSs to enhance their computation offloading performance in [26]. In [27], horizontal cooperation among edge servers and three-layer vertical cooperation were considered during task offloading. To reduce the average task duration, the offloading decisions and computing resource allocation were optimized by using the alternating direction multiplier method and difference of convex functions programming. Deep reinforcement learning was applied to achieve privacy-preserving task offloading in mobile blockchains in [28].

The above research works assumed that each edge server caches all services and could handle any type of computing task. However, it is difficult for the actual edge server to cache all services as its storage resources are limited. Therefore, it is necessary to develop a suitable service caching strategy according to the actual task requirements. Relevant research had been devoted to the edge service caching problem. In [19], service caching was used as a constraint to limit the computation offloading location of user tasks, but the service types on each edge server were fixed, which was not fitting for dynamic task requirements. An adaptive edge caching scheme based on location awareness was designed to optimize the hit rate of the caching service strategy by predicting the popularity of content in [29]. In [30], multi-dimensional features such as historical and future data information, social relationships, and geographical location were further considered to design the prevalence model and reduce prediction errors. However, it would cause all edge nodes to prefer to select popular service caching and relatively unpopular services were only solved in the cloud server, which would result in high transmission delay. The service caching strategy and task offloading policy based on the ε-greedy strategy and the Gibbs sampling principle were proposed to reduce the computing delay in [31], respectively. As the horizontal collaboration among edge servers was not taken into account, it resulted in low resource utilization among edge devices. In [32], a decentralized cooperative service placement algorithm (CSP) was proposed to improve Gibbs sampling as a service caching strategy to maximize the system utility under cellular full and non-full cooperation. However, the computing resource limitation of edge servers was not considered.

In contrast to the above works, the collaborative task offloading problem, assisted by dynamical cache strategy in MEC, is studied by considering several aspects such as collaboration, wise service caching, balanced task offloading, and fair resource allocation, which guarantees strict execution delay under the constrained computation and storage resources of edge servers.

## 3. System Model

### 3.1. Network Model

**Figure 1:**
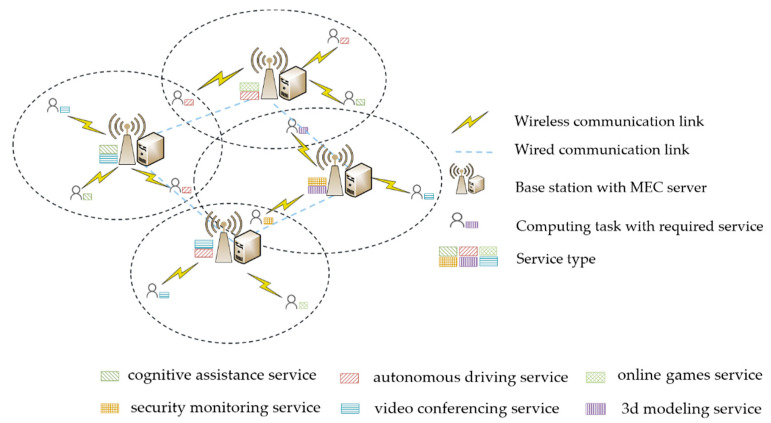
Network Model.

As shown in Figure 1, we consider a two-layer collaborative MEC network model. It consists of N mobile terminal users (TUs) and M wireless base stations (BSs). Each TU is connected to its associated BS via a wireless link, and each BS communicates with each other through a wired link. Each BS is equipped with an MEC server, serving as an edge node to provide certain computing and storage resources. The execution of each user task depends on the required service, and the type of service corresponds to the type of task. At present, emerging applications will all be used as user tasks, so the whole system includes application service types such as cognitive assistance, autonomous driving, online games, security monitoring, VR/AR, video conferencing, 3D modeling, and so on. The concept of the BS is equivalent to the MEC in the subsequent sections.

Divide the continuous time into T separate slots, where slot t represents the t-th slot. In each slot, the location of TUs and the transmission channel condition are considered fixed [33]. In order to simplify the model analysis, it is assumed that each user has only one mobile terminal, and one computing task is generated in a time slot. This task can either be processed locally or offloaded to an edge server for computing. It will be uploaded first to its associated BS if the TU performs the offloading decision, and it can be handled by its associated BS provided that there are sufficient computing resources and relevant services cached. Otherwise, the task will be further forwarded to a nearby collaborative BS with the required services and computing demands. Besides, the associated BS refers to the base station that is closest to a TU and with the best channel condition in the current time slot.

The set of BSs and TUs are denoted by M={1,2,…,M} and N={1,2,…,N}, respectively. In a slot, the TU n generates a computation task, which is given by $I_{n}=\{D_{n},\lambda_{n},S_{n},t_{n}^{\max}\}$. $D_{n}$ indicates the size of input data of the task, and $\lambda_{n}$ represents the number of CPU cycles required of the task. $S_{n}$ denotes the type of service required of the task, and $t_{n}^{\max}$ is the maximum delay limit of the task. The set of computing tasks generated by all TUs is $I=\{I_{1},I_{2},\ldots ,I_{n}\}$, and the set of service types available in the whole scenario is $S=\{S_{1},S_{2},\ldots ,S_{l}\}$. The set of TUs associated with the base station m is $N_{m}$. If user n is associated with the base station m, then $n\in N_{m}$. The main symbols and their definitions are summarized in Table 1.

### 3.2. Communication Model

Each TU is connected to its associated BS via a wireless link. At the same time, the Orthogonal Frequency Division Multiple Access (OFDMA) communication mode is used in the cell, each TU transmits its task through an orthogonal channel, so that the interference in the cell can be ignored. Besides, to simplify the problem, inter-cell interference is not considered for the time being, since interference management is not the focus of this paper. We define $R_{nm}$ as the uplink transmission rate, which is from the user n to its associated BS m. Its value depends on the number of TUs associated with the BS. Assuming that TUs connected to the same base station share communication resources equally, then $R_{nm}$ can be expressed as

(1) $$R_{nm}=\frac{W\log_{2}(1+\frac{P_{n}h_{nm}}{\sigma^{2}})}{\left|N_{m}\right|}$$

where W is the available spectrum bandwidth, $P_{n}$ and $h_{nm}$ represent the uplink transmission power and the channel gain between the user n and its associated base station, respectively. $\sigma^{2}$ is the additive Gaussian white noise power, and $\left|N_{m}\right|$ represents the number of TUs associated with the BS m.

### 3.3. Computation Offloading Model

Assume the tasks generated by each TU are inseparable, and they are supposed to be executed locally, offloaded to its associated BS, or further offloaded to a collaborative BS for computation. Define $X=\left\{x_{mk,I_{n}}|m\in ℳ,k\in ℳ\cup \left\{0\right\},n\in N_{m}\right\}$ as the task offloading strategy for the system. $x_{mk,I_{n}}\in \left\{0,1\right\}$ is the offloading decision variable for the user n, where $x_{mk,I_{n}}=1$ indicates the user task $I_{n}$ associated with m is executed by k, otherwise, $x_{mk,I_{n}}=0$. In addition, k=0 indicates $I_{n}$ is performed locally, k=m indicates $I_{n}$ is executed by its associated BS m, and k∈ℳ\{m} indicates $I_{n}$ is calculated by a non-associated collaborative BS k. The task offloading decision should satisfy

(2) $$\sum_{k\in ℳ\cup \left\{0\right\}}x_{mk,I_{n}}=1,\forall m\in ℳ,n\in N_{m}$$

#### 3.3.1. Local Computing

Assume the computing capability (i.e., the CPU cycles per second) of user n is denoted by $f_{n}^{L}$. Accordingly, the local computing delay of the task $I_{n}$ can be expressed as

(3) $$T_{n}^{L}=\frac{\lambda_{n}}{f_{n}^{L}}$$

#### 3.3.2. Associated Base Station Computing

If a TU executes one task on its associated BS, then the whole offloading delay includes three parts: the uploading time $T_{nm}^{tr}=D_{n}/R_{nm}$, the computing time $T_{nm}^{exe}$ in associated BS m, and the downloading delay of computation results. Since the computation results are usually much smaller than the input data and the downlink transmission rate is very high, we ignore the last part of the downloading delay [18]. Besides, we define the computing resource allocation strategy of the edge server as $ℱ=\left\{f_{mn}|m\in ℳ,n\in N_{m}^{exe}\right\}$, where $f_{mn}$ represents the computing resources allocated by edge server m to user n, $N_{m}^{exe}$ represents a set of tasks performed by m. The tasks in $N_{m}^{exe}$ include hit by its local cache and offloaded by other collaborative BSs. Due to the limited computing capabilities of edge servers, the resources allocated to users cannot exceed their total resources, which must be satisfied $\sum_{n\in N_{m}^{exe}}f_{mn}\leq f_{m}$. In this case, the computing time of the associated BS is $T_{nm}^{exe}=\lambda_{n}/f_{mn}$. Consequently, the total execution delay in the associated BS m can be expressed as

(4) $$T_{nm}=T_{nm}^{tr}+T_{nm}^{exe}=\frac{D_{n}}{R_{nm}}+\frac{\lambda_{n}}{f_{nm}}$$

#### 3.3.3. Non-Associated Collaborative Base Station Computing

The calculation time in a non-associated collaborative BS includes four parts: the uploading time $T_{nm}^{tr}$, the transmission time $T_{mk}^{tr}$ from the associated BS m to the collaborative BS k, the computing time $T_{nk}^{exe}$ in k, and the ignorable downloading delay. Define the transmission rate between m and k as a fixed value $R_{mk}$, then $T_{mk}^{tr}=D_{n}/R_{mk}$. According to the computing resource allocation strategy, the computing resources allocated by the collaborative BS k to the user n are $f_{kn}$, then $T_{nk}^{exe}=\lambda_{n}/f_{kn}$. Therefore, the total execution delay in the non-associated collaborative BS k can be expressed as

(5) $$T_{nk}=T_{nm}^{tr}+T_{mk}^{tr}+T_{nk}^{exe}=\frac{D_{n}}{R_{nm}}+\frac{D_{n}}{R_{mk}}+\frac{\lambda_{n}}{f_{kn}}$$

### 3.4. Service Caching Model

Only when the relevant application services are cached in advance can the corresponding computing tasks be executed by the edge server. We define the service caching strategy of the edge server as $C=\left\{c_{m,s}|m\in ℳ,s\in S\right\}$. $c_{m,s}$ is the service caching decision variable for server m, where $c_{m,s}=1$ indicates the server m caches the service s, otherwise, $c_{m,s}=0$. Due to the limited storage resources of the MEC server, the total amount of services cached by each MEC cannot exceed its capacity. Therefore, we have the following caching decision constraint

(6) $$\sum_{s\in S}c_{m,s}D_{s}\leq K_{m},\forall m\in ℳ$$

where $D_{s}$ is the data size of service s, $K_{m}$ is the storage capacity of edge server m.

### 3.5. Service Caching Model

A TU generates one computing task in a time slot, which can optionally be executed locally or offloaded to its associated or collaborative BS with the required services and computing demands in advance. Assume that TUs can perform all tasks generated by themselves locally, the actual computation delay of the task $I_{n}$ is

(7) $$T_{n}=x_{m0,I_{n}}T_{n}^{L}+c_{m,s_{n}}x_{mm,I_{n}}T_{nm}+\sum_{k\in ℳ\backslash \left\{m\right\}}c_{k,s_{n}}x_{mk,I_{n}}T_{nk}$$

We develop the joint optimization problem of collaborative offloading strategy X, computation resource allocation strategy ℱ, and service caching strategy C with the consideration of user fairness, where the fairness is reflected by minimizing the maximum actual delay $T_{n}$ of all users. Accordingly, the objective problem can be formulated as

(8) $$\begin{array}{lll} P1: & & \underset{C,X,ℱ}{\min}\;\underset{n\in N}{\max}T_{n}\\ s.t. & C1: & \sum_{s\in S}c_{m,s}D_{s}\leq K_{m},\forall m\in ℳ\\ & C2: & \sum_{k\in ℳ\cup \left\{0\right\}}x_{mk,I_{n}}=1,\forall m\in ℳ,n\in N_{m}\\ & C3: & \sum_{n\in N_{m}^{exe}}f_{mn}\leq f_{m},\forall m\in ℳ\\ & C4: & f_{mn}\geq 0,\forall m\in ℳ\\ & C5: & c_{m,s}\in \left\{0,1\right\},\forall m\in ℳ,s\in S\\ & C6: & x_{mk,I_{n}}\in \left\{0,1\right\},\forall m\in ℳ,k\in ℳ\cup \left\{0\right\},n\in N_{m} \end{array}$$

where the constraint C1 indicates that the total amount of services cached by each MEC cannot exceed its capacity. C2 ensures that a TU can only perform at one of its local, associated BS, or collaborative BS. C3 denotes that the total computation resources allocated by an MEC cannot exceed its computing capability. C4 means the computation resources allocated are non-negative. C5 represents that the service caching decision is a binary variable and it can only be service cached or not cached. C6 represents that the task offloading decision is a binary variable and it can only be task offloaded or not offloaded.

## 4. Joint Optimization Strategy of Task Offloading and Service Caching

In this section, an efficient computation offloading strategy called JTOSC is proposed to achieve the goal of P1. Since the service caching and task offloading variables are 0 or 1, the computation resources allocation result can be any value between 0 and 1. Therefore, problem P1 is a mixed integer nonlinear programming problem. In addition, $c_{m,s}$ and $x_{mk,I_{n}}$, $x_{mk,I_{n}}$ and $f_{mn}$ are coupled with each other, leading to the objective function being non-convex and difficult to tackle. Thus, we decompose P1 into two sub problems to solve, namely service caching and task scheduling problem, where the task scheduling problem can be further divided into task offloading decision and fair resource allocation.

### 4.1. Service Caching Model

In the outer layer of JTOSC, the service caching decision of MEC is determined iteratively based on Gibbs sampling, where the main idea of Gibbs sampling is to simulate conditional samples by scanning each variable while keeping the remaining variables constant in each iteration. Specifically, the update process of service caching decision is regarded as a L dimensional Markov chain. In each round of iteration, an edge server m∈ℳ and a feasible caching strategy $C_{m}^{*}\in C$ satisfying the relevant constraints are randomly selected, while the caching strategies on the remaining edge servers maintain unchanged. Based on the caching decisions of all edge servers in the previous round and the current round, the task offloading strategy X and $X^{*}$, the computing resource allocation strategy ℱ and ${ℱ}^{*}$, the objective function value τ and $\tau^{*}$ can be calculated for the previous round and the current round, respectively. Associate the conditional probability distribution of cache update strategies with the optimization goal of P1, accepting the current caching strategy with probability ρ, and maintaining the previous round of caching strategy with probability 1−ρ. Eventually, the Markov chain will converge to the optimal caching policy with high probability. The service caching strategy is shown in Algorithm 1.
**Algorithm 1:** Service Caching Algorithm based on Gibbs Sampling**Input:**N, ℳ, S, $D_{s}\left(s\in S\right)$, $K_{m}(m\in ℳ)$, w**Output:**C, X, ℱ, τ, $\tau^{ave}$1: Initialize $C^{0}\leftarrow 0$, L
2: for l=1:L do3: Randomly select an MEC server m∈ℳ and a feasible caching strategy $C_{m}^{*}\in C$;4: Based on the previous round caching strategy $\left\{C_{1}^{l-1},C_{2}^{l-1},\ldots C_{M}^{l-1}\right\}$, compute the task offloading strategy X and resource allocation strategy ℱ and objective function value τ and $\tau^{ave}$;5: Based on the current round caching policy $\left\{C_{1}^{l},C_{2}^{l},\ldots C_{m}^{*},\ldots C_{M}^{l}\right\}$, compute the task offloading strategy $X^{*}$ and resource allocation strategy ${ℱ}^{*}$ and objective function value $\tau^{*}$ and $\tau^{*ave}$;6: Let $C_{m}^{l}=C_{m}^{*}$ with the probability $\rho =\frac{1}{1+e^{\left(\tau^{*}-\tau \right)/w}}$;7: Let $C_{m}^{l}=C_{m}^{l-1}$ with the probability 1−ρ;8: end for

When the outer layer service caching decision is determined, the original optimization problem P1 is reduced to the inner layer task scheduling problem P2.

(9) $$\begin{array}{lll} P2: & & \underset{X,ℱ}{\min}\;\underset{n\in N}{\max}T_{n}\\ s.t. & & C2,C3,C4,C6 \end{array}$$

In optimization problem P2, the task offloading strategy X is coupled with the computation resource allocation strategy ℱ, where ℱ depends on the result of X, and X needs to be further adjusted and optimized according to the result of ℱ. We consider solving these two coupled problems alternatively by fixing one of the result terms.

### 4.2. Computing Resource Allocation Based on Fairness Perception

We define the fairness of TUs from the perspective of user experience, which can be reflected by minimizing the maximum actual delay $T_{n}$ of all users. Specifically, we propose a fairness perception computing resource allocation strategy, fairly allocating all computing resources to TUs. By initializing the task offloading decision X, P2 is simplified to the computing resource allocation problem P3 as follows:

(10) $$\begin{array}{lll} P3: & \underset{ℱ}{\min} & \underset{n\in N_{off}}{\max}\sum_{k\in ℳ}c_{k,s_{n}}x_{mk,I_{n}}\frac{\lambda_{n}}{f_{kn}}+Q_{n}\\ s.t. & C3^{\prime }: & \sum_{n\in N_{k}^{exe}}f_{kn}\leq f_{k},\forall k\in ℳ\\ & C4^{\prime }: & f_{kn}\geq 0,\forall k\in ℳ \end{array}$$

given the service caching decision and the task offloading decision, the second term $Q_{n}$ in P3 is a fixed value, and its value can be clearly expressed as $Q_{n}=x_{m0,I_{n}}\lambda_{n}/f_{n}^{L}+c_{m,s_{n}}x_{mm,I_{n}}D_{n}/R_{nm}+\sum_{k\in ℳ\backslash \left\{m\right\}}c_{k,s_{n}}x_{mk,I_{n}}\left(D_{n}/R_{nm}+D_{n}/R_{mk}\right)$, where $N_{off}$ is the set of all TUs offloaded to MECs, and $N_{k}^{exe}$ is the set of TUs offloaded to MEC k.

Meanwhile, since both caching decision and offloading decision are binary variables, and only one of the offloading decision variables ($x_{m0,I_{n}}$, $x_{mm,I_{n}}$ and $x_{mk,I_{n}}$) is equal to 1, let $\sum_{k\in ℳ}c_{k,s_{n}}x_{mk,I_{n}}\lambda_{n}/f_{kn}+Q_{n}=\lambda_{n}/f_{kn}+Q_{n}\leq \tau$. At this time

(11) $$\tau =\begin{array}{cc} \underset{n\in N_{off}}{\max}\sum_{k\in ℳ}c_{k,s_{n}}x_{mk,I_{n}}\frac{\lambda_{n}}{f_{kn}}+Q_{n} & \end{array}$$

where $\lambda_{n}/f_{kn}$ is the computation delay of MEC k, and its value is non-negative. Then, $0\leq \lambda_{n}/f_{kn}\leq \tau -Q_{n}$. This constraint of $f_{kn}$ can be transformed into $0\leq \lambda_{n}/\left(\tau -Q_{n}\right)\leq f_{kn}$. MEC k allocates computing resources to all offloaded users in $N_{k}^{exe}$, and the sum can be obtained.

(12) $$\sum_{n\in N_{k}^{exe}}\frac{\lambda_{n}}{\tau -Q_{n}}\leq \sum_{n\in N_{k}^{exe}}f_{kn}\leq f_{k}$$

Only when we put all computing resources to work can we ensure that each TU is allocated relatively more computing resources from MEC and obtain higher quality performances. Therefore,

(13) $$\sum_{n\in N_{k}^{exe}}\frac{\lambda_{n}}{\tau -Q_{n}}=\sum_{n\in N_{k}^{exe}}f_{kn}=f_{k}$$

At this point, the problem of computing resource allocation is transformed into

(14) $$\begin{array}{ll} P3^{\prime }: & \underset{ℱ}{\min}\tau \\ s.t. & C7^{\prime }:\sum_{n\in N_{k}^{exe}}\frac{\lambda_{n}}{\tau -Q_{n}}=\sum_{n\in N_{k}^{exe}}f_{kn}=f_{k},\forall k\in ℳ \end{array}$$

where the constraint $C7^{\prime }$ is a monotonically decreasing function of τ, $\tau_{\min}=Q_{n}$ and $\tau_{\max}=\sum_{n\in N_{k}^{exe}}(\lambda_{n}/f_{k}+Q_{n})$. Use the bisection method to calculate the optimal objective function value τ within the upper and lower bounds. The computing resource allocation process is shown in Algorithm 2.
**Algorithm 2:** Computing Resource Allocation based on Fairness Perception**Input:** C, X, tolerance ξ**Output:** ℱ, τ, $\tau_{ave}$1: for k∈ℳ do2: for $n\in N_{k}^{exe}$ do3:    $\tau_{\min}=Q_{n}$;4:    $\tau_{\max}=\sum_{n\in N_{k}^{exe}}\left(\frac{\lambda_{n}}{f_{k}}+Q_{n}\right)$;5:  while $\left|\tau_{\max}-\tau_{\min}\right|\geq \xi$
6:    $\tau_{mid}=\frac{\tau_{\max}-\tau_{\min}}{2}$;7:    if $\sum_{n\in N_{k}^{exe}}\frac{\lambda_{n}}{\tau_{mid}-Q_{n}}\geq f_{k}$
8:       $\tau_{\min}=\tau_{mid}$;9:    else10:     $\tau_{\max}=\tau_{mid}$; 11:     end if12:  end while13:  $\tau_{n}=\tau_{\min}$;14: end for15:  $ℱ\leftarrow f_{kn}\leftarrow \tau_{n}$, according to Equation (14);16: end for17:  $\tau_{\max}=\underset{n\in N_{off}}{\max}\left\{\tau_{n}\right\}$;18:  $\tau_{ave}=\frac{\sum_{n\in N_{off}}\tau_{n}}{\left|N_{off}\right|}$;

### 4.3. Bilateral Matching Task Offloading Based on Revenue Preference

In the previous section, a fixed task offloading strategy was used to allocate computing resources. However, it is necessary to continuously adjust the offloading scheme according to a reliable offloading strategy. At this point, the optimization problem is transformed into:

(15) $$\begin{array}{ll} P4: & \underset{X}{\min}\;\underset{n\in N}{\max}T_{n}\\ s.t. & C2,C6 \end{array}$$

where the value of $T_{n}$ is given in Equation (7).

The set of BSs that cache the services required by the task $I_{n}$ is defined as ${ℳ}_{n}^{candidate}$. The locations where the task can be executed include the local TU and MEC m, satisfying $\forall m\in {ℳ}_{n}^{candidate}$. Each TU sends the offloading request to its own associated BS at the beginning of a time slot, and the set of offloading requests received by the associated BS is defined as $N_{m}^{req}$, which includes the tasks offloaded by the associated TUs and the collaborative BSs. If the associated BS m belongs to ${ℳ}_{n}^{candidate}$, that is, its local cache hits the service required by the task $I_{n}$. Then, these tasks hit will be added to $N_{m}^{candidate}$, and the missed will be added to the set $N_{m}^{no}$. The initial task offloading scheme assumes that all tasks in $N_{m}^{candidate}$ are executed by MEC m, each task in $N_{m}^{no}$ sends its offloading request to collaborative BSs with the highest preference value in ${ℳ}_{n}^{candidate}$, and the collaborative BS executes all tasks received. Meanwhile, the computing resources allocation strategy of TUs is computed by Formula (14). So far, the initial service caching, task offloading, and computing resource allocation scheme are obtained.

With the updated service caching decision, the task offloading strategy adopts a preference-based bilateral matching algorithm to select the appropriate offloading location. Calculate the objective function value $T_{n}$ of each TU under the current offloading decision. If all TUs meet their maximum delay requirements and do not exceed the computing resources constraint of each BS, then the offloading scheme at this time is suitable. Otherwise, define the difference between the task maximum latency limit and its actual latency as the task offloading revenue, that is $\gamma_{nm}=t_{n}^{\max}-T_{n}$. Calculate the offloading revenue of each TU in $N_{m}^{exe}$, and select the task with the smallest revenue in turn for further offloading. Then, remove it from $N_{m}^{exe}$ to $N_{m}^{off}$, until all the remaining tasks in $N_{m}^{exe}$ can meet the maximum delay and computing resources constraints. So far, we obtain the set $N_{m}^{exe}$ of user tasks calculated by the associated BS, and the set $N_{m}^{off}$ of user tasks rejected by the associated BS and need to be further offloaded.

For each TU in $N_{m}^{off}$, a preference-based approach is adopted to select an appropriate offloading location. Each task to be further offloaded has a preference for different offloading locations, and the preference value is related to the estimated delay of the offloading location. The larger the estimated offloading delay, the smaller the preference value. In this case, the task $I_{n}$ is rejected by the MEC m and needs to be further offloaded has a preference value for the collaborative BS k, which can be expressed as

(16) $$x_{m,I_{n}}\left(k\right)=\frac{1}{\frac{D_{n}}{R_{nm}}+\frac{D_{n}}{R_{mk}}+\frac{\lambda_{n}}{f_{kn}}},\forall m\in ℳ,k\in {ℳ}_{n}^{candidate}$$

The task $I_{n}$ that is rejected by the edge device m and needs to be further offloaded has a preference value for its local TU, which can be expressed as

(17) $$x_{m,I_{n}}\left(0\right)=\frac{1}{\frac{\lambda_{n}}{f_{n}^{L}}}$$

The task $I_{n}$ sends its offloading request to the location with a high preference value preferentially. If the location requested is the local TU, then the offloading request will be accepted directly, and let $x_{n0,I_{n}}=1$. If the location requested is the collaborative BS, then the BS reply is needed. If the offloading request is rejected, then it will be sent to the next best offloading location in the next iteration until it is accepted and let $x_{nk,I_{n}}=1$ at once. Repeat the above process until all offloading decisions are confirmed, then the algorithm terminates. The preference-based bilateral matching task offloading process is shown in Algorithm 3.
**Algorithm 3:** Preference-Based Bilateral Matching Offloading Algorithm**Input:** ℐ, C**Output:** X1: Initialize ${ℳ}_{n}^{candidate}$, $N_{m}^{req}$, $N_{m}^{candidate}$, $N_{m}^{no}$, $N_{m}^{rec}$, $N_{m}^{off}$, $N_{m}^{exe}$ equal to Ø;2: User side: each user sends an offloading request to its associated BS;3: MEC side: BSs mutually forward the users offloading requests;4: Initial task offloading:5: for m∈ℳ do6: $N_{m}^{req}\leftarrow$ received offloading requests;7: $N_{m}^{candidate}$, $N_{m}^{no}$$\leftarrow C_{0}$;8: Initial offloading strategy $X_{0}$: $N_{m}^{candidate}\to x_{nm,I_{n}}=1$, $N_{m}^{no}\to$ according to user preferences, with full acceptance of offloading requests;9: Initial resource allocation strategy: ${ℱ}_{0}\leftarrow X_{0}$, according to Algorithm 2;10: end for11: for n∈N do12:  Computing $T_{n}\leftarrow$ Equation (7);13:  $N_{m}^{exe}\leftarrow m\leftarrow X$;14: if $T_{n}\leq t_{n}^{\max}$ and $\underset{n\in N_{m}^{exe}}{\Sigma }f_{mn}\leq f_{m}\left(\forall m\in ℳ,\;\forall n\in N_{m}^{exe}\right)$
15:   $N_{m}^{exe}=N_{m}^{exe}$;16:   $x_{nm,I_{n}}=1$;17: else18:  Computing $\gamma_{nm}(\forall n\in N_{m}^{exe})$;19:  Sort $\gamma_{nm}$ in descending order, select a task with the smallest value to offload in turn, let $N_{m}^{exe}=N_{m}^{exe}\backslash \left\{n\right\}$ and $N_{m}^{off}=N_{m}^{off}\cup \left\{n\right\}$ until and $\sum_{n\in N_{m}^{exe}}f_{mn}\leq f_{m}$$\left(\forall n\in N_{m}^{exe}\right)$;20: end if21: end for22: for $n\in N_{m}^{off}$ do23: Computing offloading preference $x_{m,I_{n}}\left(k\right)$($\forall k\in {ℳ}_{n}^{candidate}$);24: Sort $x_{m,I_{n}}\left(k\right)$ in descending order, select a collaborative BS k with the biggest value to send the offloading request preferentially.25: if k=0 do26:  Accept the offloading request of $I_{n}$, let $N_{m}^{off}\backslash \left\{n\right\}$, $x_{n0,I_{n}}=1$;27: else28:  if k accepts the offloading request of $I_{n}$, let $N_{m}^{off}\backslash \left\{n\right\}$, $x_{nk,I_{n}}=1$;29:  else send the offloading request of $I_{n}$ to the suboptimal collaborative BS k, until it is accepted, let $x_{nk,I_{n}}=1$;30:  end if31: end if32: end for

### 4.4. Complexity Analysis

The outer layer in JTOSC iteratively updates the service caching decisions based on Gibbs sampling. Its time complexity is O(L), where L represents the number of iterations for the outer layer of proposed algorithm. The inner layer in JTOSC adopts the fairness-aware computing resources allocation algorithm for MEC servers. With a precision ξ and an initial interval $\left(\tau_{\max}-\tau_{\min}\right)$, the resource allocation algorithm can be resolved by the bisection method within $O(\log_{2}\frac{\tau_{\max}-\tau_{\min}}{\xi })$ iterations. Let $N_{1}=\left|N_{m}^{exe}\right|$ to represent the set of TUs executed by the MEC m. Considering there are M MEC servers, the complexity of resource allocation for a task offloading scheme is $O(M\times N_{1}\times \log_{2}\frac{\tau_{\max}-\tau_{\min}}{\xi })$. Eventually, the time complexity of our proposed JTOSC iterative algorithm is the product of internal and external code complexity, that is $O(L\times M\times N_{1}\times \log_{2}\frac{\tau_{\max}-\tau_{\min}}{\xi })$.

## 5. Simulation Results and Performance Analysis

### 5.1. Simulation Setting

Considering the edge computing scenario where four BSs and many users are randomly distributed, each BS is deployed with an MEC server. The system bandwidth is set to 20 MHz, and the background noise power is −100 dBm. The path loss factor used in this paper refers to the setting of [17], i.e., $L\left[dB\right]=140.7+36.7\log_{10}d[km]$. For computing tasks, we consider face detection and recognition applications for airport security and surveillance, and they can benefit from collaboration between TUs and the MEC platform [34]. In most simulations, unless otherwise specified, we consider the number of user tasks as 20, the input size of the task to be set to $D_{n}=420$ KB, the number of CPU cycles required of task to be set to $\lambda_{n}=1000$ Megacycles, and the computing capability of MEC as 20 GHz. Assume they contain six types of services, which satisfies all task requirements in system. Simulation is performed on MATLAB to evaluate the performance of the proposed joint optimization strategy of task offloading and service caching. The main simulation parameters are listed in Table 2.

### 5.2. Strategies Comparison

In order to better evaluate the performance of the proposed strategy, we compared it with the following four task offloading strategies.

(1) Computation Offloading and Resource Allocation algorithm (CORA) [18]. Tasks generated by TUs are calculated locally or by the cloud, and the edge servers do not cache any services;
(2) Joint Task Offloading and Resource Allocation algorithm (JTORA) [17]. Task offloading and resource allocation in a multi-users and multi-severs scenario is optimized without considering MEC collaboration, using the caching strategy in this paper for service caching;
(3) Optimizing Service Placement and Resource Allocation algorithm (OSPRA) [13]. Service placement and resource allocation are optimized without considering MEC collaboration, using service popularity to greedy cache relatively more popular services;
(4) Collaborative Data Caching and Computation Offloading (CDCCO) [14]. MEC collaborates with each other for task offloading, and we adopt the dynamic programming algorithm that caches data in the original algorithm for service caching.

The performance of each strategy is evaluated by four indicators: the maximum execution delay of all users, the average execution delay, the number of load tasks, and the local service caching hit ratio of each edge server. The local service caching hit ratio refers to the ratio of hit services number to required services number about the associated BS and its users.

### 5.3. Analysis of Simulation Results

**Figure 2:**
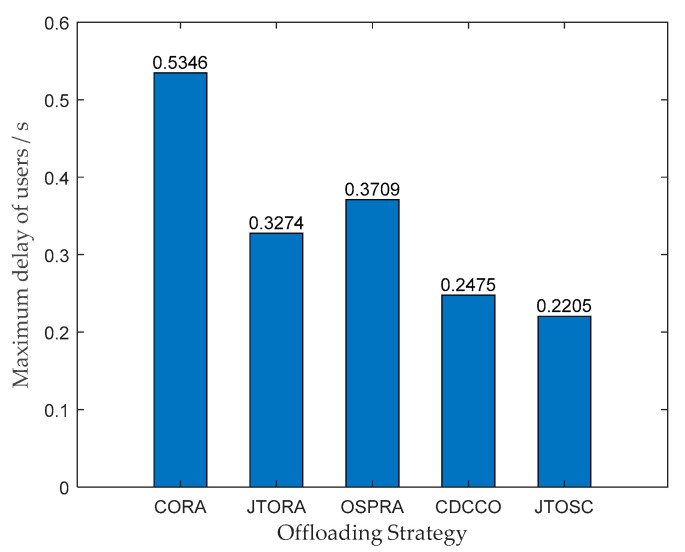
The maximum delay of users under different strategies.

In Figure 2, the maximum delay of all users, which reflects user fairness sideways, is compared. It can be seen from Figure 2 that TUs generate the largest delay when choosing the CORA strategy because of the weak computing capability of TUs themselves and the far distance between TUs and the cloud, leading to high execution delay and transmission delay, respectively. Compared with the CORA strategy, the tasks can be offloaded to MEC servers, which brings more resources and closer distance. Hence, the maximum delay of all users of the other four strategies was cut down as a result.

Simultaneously, the JTOSC and CDCCO strategies show better performance than the JTORA and OSPRA strategies—the reason is whether to consider the collaboration between MECs. The tasks not hit locally can be offloaded to the collaborative MECs satisfying demands preferentially rather than the remote cloud directly, which reduces the transmission delay and balances the edge load. Besides, the JTOSC and JTORA use an iteratively updated strategy based on probability in this paper to perform service caching, better than the dynamic programming cache in CDCCO and the greedy cache in OSPRA. Therefore, the JTORA strategy shows slightly better performance than the OSPRA, and the JTOSC strategy displays the most excellent performance.

**Figure 3:**
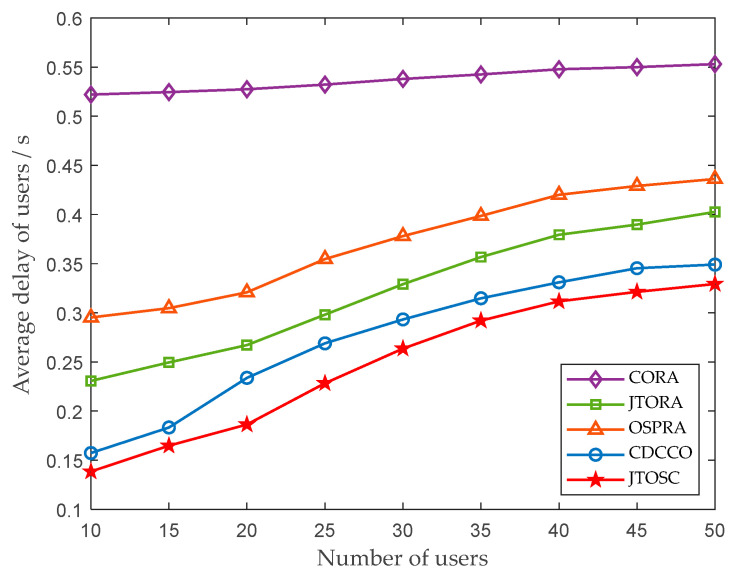
The impact of the number of users on the average delay of users.

In Figure 3, the impact of the different numbers of users on the average delay of tasks is illustrated, where the average delay is the overall tasks delay divided by the number of tasks executed. With the increasing number of users, the average delay of all tasks presents an upward trend. Increasing users lead to intensified communication competition among them, then in turn raises the delay slightly in the CORA strategy. Meanwhile, due to the constrained resources of MECs, queuing and further offloading cause redundant waiting and transmission delay, respectively, in the other four strategies, leading to more overall delay and average delay. From Figure 3, it can be concluded that the CDCCO and JTOSC strategies show better performance. As there are more computing resources for task offloading because of the MECs’ collaboration, the delay is relatively reduced.

**Figure 4:**
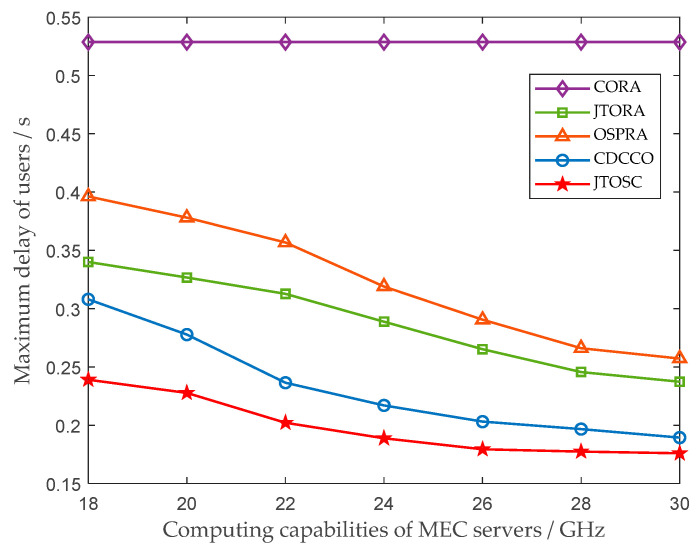
The impact of the computing capabilities of MEC servers on the maximum delay of users.

In Figure 4, the impact of the computing capabilities of MEC servers on the maximum delay of all users is illustrated. The improvement of the computing capabilities does not have any influence on the CORA strategy, since its edge servers do not cache computing services and cannot participate in computing any user tasks. In the remaining four strategies, with the computing capabilities of edge servers increasing, the computing resources allocated to user increase, then the computing delay decrease. However, due to the limitation of storage resources of edge servers, they are unable to cache more services to perform more tasks, so the downward trend gradually stabilizes. In addition, it can be visualized from Figure 4 that the performance difference between MECs’ non-collaboration (OSPRA and JTORA) and collaboration (CDCCO and JTOSC) strategies gradually decreases. This is because that the number of user tasks, which can be processed by the associated MEC itself, increases with the greater computing capabilities.

**Figure 5:**
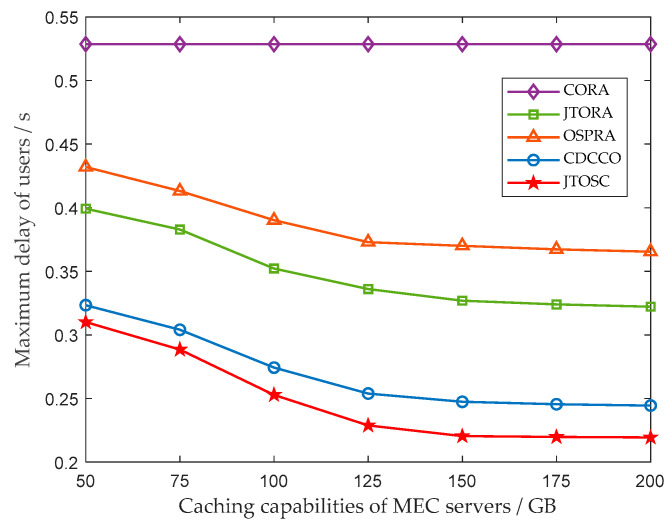
The impact of the caching capabilities of MEC servers on the maximum delay of users.

In Figure 5, the impact of the caching capacities of MEC servers on the maximum delay of all user tasks is illustrated. Similarly, the increase of the storage capacities of edge servers does not affect the maximum delay of all users, since the edge servers cannot participate in computing any user tasks in the CORA strategy. In the remaining four strategies, with the storage capacities of the edge servers increasing, the services required will be cached with a greater probability, reducing further offloading to collaborative MECs and remote cloud, and the maximum delay decreases with it. Moreover, it can be seen from Figure 5 that the downward trend gradually becomes stable while the caching capacity reaches about 125 GB. This means that the edge servers are limited mainly by their own computing resources at this time.

**Figure 6:**
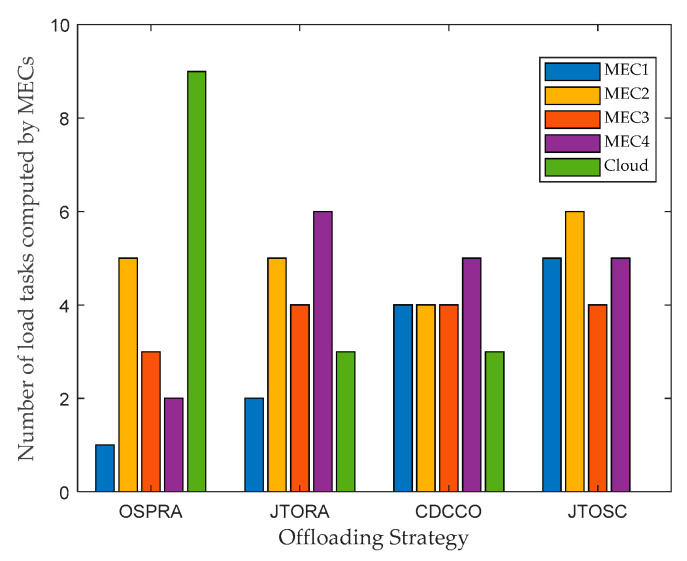
Comparison of the number of load tasks computed by MECs with different strategies.

In Figure 6, the comparison of the number of load tasks executed by each edge server and cloud under four strategies is illustrated. The CORA strategy is not compared, since all tasks will be offloaded to the remote cloud for execution under CORA. Both the OSPRA and JTORA strategies do not consider the horizontal collaboration between edge servers, resulting in an unbalanced load among MECs. On the contrary, the CDCCO and JTOSC strategies consider the horizontal collaboration among MECs, and their loads are relatively balanced. Besides, the number of tasks performed by each edge server is related to its own service cache hit rate. Most tasks were performed by MECs in JTOSC because of its better iteratively update service caching strategy.

**Figure 7:**
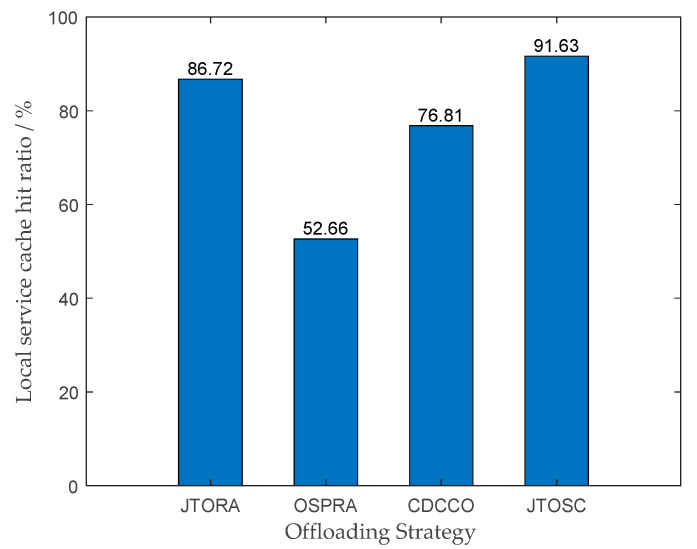
Comparison of the local service cache hit ratio under different strategies.

In Figure 7, the comparison of the local service cache hit ratio of edge servers under four strategies is illustrated. Similarly, the CORA strategy does not participate in the comparison. As we can see, the JTOSC strategy proposed in this paper possesses the highest hit ratio, and the second one is the JTORA, indicating that the performance of the proposed caching strategy is excellent. The dynamic programming method for caching in CDCCO is better than the greedy cache in OSPRA. Because the greedy cache preferentially chooses popular services, relatively unpopular services can only be stored in the cloud, resulting in high transmission delay.

## 6. Conclusions

In this paper, a collaborative task offloading problem assisted by dynamical service caching in MEC is investigated to reduce the maximum delay of all users by jointly considering the service caching decisions, task offloading decisions, and computing resource allocation. A service caching strategy based on Gibbs sampling is proposed to select appropriate services for computing. Furthermore, a computing resources allocation strategy based on fairness is presented to improve the equity among users certainly. Moreover, an offloading revenue preference-based bilateral matching strategy is introduced for offloading location options. The simulation results have demonstrated that the proposed JTOSC can effectively reduce the maximum delay of all users, improve the user experience, and balance the edge load. In this work, it is assumed that all users share communication resources equally, and the inter-cell interference is ignored. Communication interference management will be studied in the next research work. This study can be reviewed as a reference for task offloading in MEC.

## Figures and Tables

**Table 1:** Parameter Notation.

Symbol	Definition
M	Base stations set
N	Users set
S	Service types set
X $,\;x_{mk,I_{n}}$	Task offloading strategy and Decision variable
C $,\;c_{m,s}$	Service caching strategy and Decision variable
F $,\;f_{mn}$	Computing resource allocation strategy and Decision variable
$D_{n}$	$\mathrm{Input}\;\mathrm{data}\;\mathrm{size}\;\mathrm{of}\;\mathrm{task}\;I_{n}$
$\lambda_{n}$	$\mathrm{CPU}\;\mathrm{cycles}\;\mathrm{required}\;\mathrm{of}\;\mathrm{task}\;I_{n}$
$S_{n}$	$\mathrm{Service}\;\mathrm{type}\;\mathrm{required}\;\mathrm{of}\;\mathrm{task}\;I_{n}$
$t_{n}^{\max}$	$\mathrm{Maximum}\;\mathrm{delay}\;\mathrm{limit}\;\mathrm{of}\;\mathrm{task}\;I_{n}$
$f_{n}^{L}$	Local computing capability of user n
$f_{m}$	Computing capability of MEC m
$f_{mn}$	Computing resources allocated by MEC m to user n
$D_{s}$	Data size of service s
$K_{m}$	Storage capacity of MEC m
$R_{nm}$	Uplink transmission rate between user n and MEC m
$R_{mk}$	Transmission rate between MEC m and k
$T_{n}^{L}$	Task local computation time
$T_{nm}^{tr}$	Task uploading time to MEC m
$T_{mk}^{tr}$	Task transmission time between MEC m and k

**Table 2:** Main Simulation Parameters.

Parameters	Value
Number of users	[10, 50]
Number of BSs	4
Number of service types	6
System bandwidth	20 MHz
User transmitting power	20 dBm
Path loss	$140.7+36.7\log_{10}d[km]$ dB
Background noise power	−100 dBm
Input data size of one task	420 KB
CPU cycles required of one task	1000 Megacycles
Maximum delay limit of one task	1.5 s
Local computing capability of user	1 GHz
Computing capability of MEC	20 GHz
The transmission rate between BSs	500 Mbps
Data size of one service	[30, 80] GB
Storage capacity of one MEC	[50, 200] GB
Smoothing parameter	$10^{-6}$

## Data Availability

Not applicable.
